# Client, caregiver, volunteer, and therapist views on a voluntary supported group exercise programme for older adults with dementia

**DOI:** 10.1186/s12877-020-01632-6

**Published:** 2020-07-08

**Authors:** Kristin Taraldsen, Elisabeth Boulton, Jorunn L. Helbostad, Ingvild Saltvedt, Randi Granbo

**Affiliations:** 1 Department of Neuromedicine and Movement Science, Faculty of Medicine and Health Sciences, Norwegian University of Science and Technology (NTNU), 7491 Trondheim, Norway; 2 Clinic of Clinical Service, St Olav University Hospital, Trondheim, Norway; 3 School of Health Sciences, Faculty of Biology, Medicine and Health, University of Manchester, Manchester, UK; 4 Department of geriatrics, St Olav University Hospital, Trondheim, Norway

**Keywords:** Elderly, Group exercise, Physical activity, Physical therapy, Dementia

## Abstract

**Background:**

Existing group exercise programmes, or other services offered to maintain physical activity levels, are typically not developed specifically for older adults with dementia. The aim of this study was to gain knowledge about perceptions of a newly developed volunteer supported group exercise programme for older adults with dementia, and any barriers that may have affected participation and compliance.

**Methods:**

Seven (six face-to-face and one by email) interviews were conducted with (i) older adults and volunteers participating in a pilot 12-week group exercise programme, (ii) caregivers, and (iii) therapists leading the group sessions. Interview transcriptions were systemised by use of NVivo 8 and analysed by use of Systematic Text Condensation method.

**Results:**

The theme “building relationships” represents the reason why attending this group was important for the participants. The findings suggest that how we organize exercise groups is important, with some sort of extra support, to ensure that persons will begin and continue to participate in new activities outside their homes.

**Conclusions:**

This study showed that it is possible to involve home-dwelling persons with cognitive decline and dementia in group exercise sessions. The role of building relationships was the major factor for successful participation. Providing support and ensuring motivation for persons attending the group outside their homes was essential, both for them and their caregivers. Service providers should not underestimate the importance of building relationships between persons involved in service offers.

## Background

Dementia is an increasing public health challenge where prevalence is strongly associated with age, and numbers will double over coming decades [1]. Dementia causes impairment in memory and other cognitive abilities including “executive function” [2], and taking part in activities of daily living (ADL) may be a challenge due to difficulties in, for example, planning, judgement and decision-making. Because of changes in physical health and cognitive function, individuals with dementia may need extra support on both an individual level and an environment level, to be able to access and participate in new activities [3].

There is an urgent need to identify or develop interventions that reduce or delay the dependency in ADL caused by dementia. One potential strategy is physical activity (PA), where the effects on health and function are well documented [4]. Exercise, in terms of structured physical activity, has also shown promising effects on ADL in older people with dementia [5], which is highly relevant to delay the dependency in daily life resulting from dementia, and maintain the ability to live at home as long as possible after being diagnosed. Furthermore, fall prevention exercise interventions are suggested highly relevant for persons with dementia, and are included in recent initiatives [6–8].

Current recommendations for older adults suggest 150 min of PA per week [9, 10], and few older adults are following the PA guidelines [11]. Furthermore, intervention studies aiming to evaluate effects of PA and exercise often do not focus on how to deliver services for older adults with cognitive decline or dementia, where one great challenge is participation. Effects of exercise interventions for older populations are limited by low uptake [12, 13]. The challenge for clinicians is to know how to deliver and motivate older persons to participate and adhere to such interventions. For older adults with declines in cognitive function, this could be even more crucial [3]. A study describing attitudes among older adults with cognitive challenges highlights the respect for the older person’s identity as crucial, and that individualised interventions, which focus on maintaining independence and preserving quality of life, may be more acceptable [14]. Thus, there is an urgent need for developing effective PA strategies for home-dwelling older adults with dementia.

To develop new services for this target group, understanding both participants’ and their caregivers’ experience of group activities outside the home, is essential. Thus, the aim of the present study was to evaluate the delivery of a volunteer-supported group exercise programme for community-dwelling older adults with cognitive decline and dementia. To get in-depth knowledge about important factors that can facilitate participation, our research question was: What are the experiences of people attending the group, their caregivers, volunteers, and those leading the group sessions, after taking part in a 12-week group exercise programme?

## Methods

### Design

A qualitative research approach using semi-structured interviews was undertaken. Qualitative methods can increase the understanding of subjective experiences and provide more in-depth information about important factors for attending group activities outside the home [15]. We aimed to evaluate experiences among participants in a 12-week, group-based exercise service, delivered once a week, to community-dwelling older adults with dementia; their caregivers; volunteers that were part of the organisation of the service; and the two physiotherapists leading the group sessions.

### Development of a new group exercise programme

Prior to the present study, we performed a cross-sectional study to describe physical function, activity levels, and participation in activities outside the home in a group of 100 home-dwelling older adults with cognitive decline or dementia. We also performed a qualitative study to focus on caregivers’ perspectives on how to design new PA interventions for home-dwelling older adults with dementia [16]. Our main findings in these two studies were that older people with cognitive decline and dementia do not take part in many activities outside their homes; their support network is limited; and the caregivers often have a comprehensive, demanding role as caregivers. New services should include strategies for how to support the person and their caregivers, so the person with dementia wants to take part and so that caregivers think that it is worth the required effort to motivate and support participation [16].

Our starting point was an existing fall prevention group-based exercise programme for independent older home-dwelling people, with exercise classes once a week focusing on balance and strength activities [17–19]. In Norway, as well as in other European countries, the use of volunteers in delivery of different services is common. To ensure that people with dementia could take part, we involved volunteers from the municipality to support the participants both to get to and from the class, and during the exercise sessions. We, two researchers from the university in collaboration with physiotherapists from the municipality, developed the content of the exercise programme based on the existing fall prevention exercise programme, but put extra emphasis on the motivational element, so that the participants enjoyed the exercise class and experienced mastery of the activities. Examples of motivating factors are including games and addressing people by their names in the warm up, as well as instructors focusing on engagement through the principle “having fun while exercising”. The latter was based on the input received in a previous study, where caregivers highlighted the importance of developing meaningful and high quality offers for our target group [16].

The group activity was offered during a 12-week period, with two-hour sessions including the one-hour programme once a week in a fitness room, located in the city centre. The design of the volunteer-supported group exercise programme is presented at a glance in Table 1. Two physiotherapists led the group sessions, where the participants trained together with their volunteer. Training of volunteers prior to joining the project included information about typical challenges due to reduced cognitive functions. As volunteers in this study, guidance and recommendations on strategies were provided, but volunteers were not limited to specific ‘rules’ on how to support participants during the exercise sessions.

**Table 1:** Design of the group exercise programme

Group-based exercise	Training of the volunteers
12 weeks of group-based exercise, once a week	2 day course
Principles: *Warm-up* (5–15 min): Starting seated continuing in standing; exercises for ankles, pelvis, shoulders and back. Throwing a ball and playing a game, including saying the names of all participants. *Endurance* (15–25 min): Walking (toe/heel walking, backwards) and change of speed. Exercise for large muscle groups in standing. *Muscle strength* (15–25 min): Squat, toe rise, lunge. *Balance* (15–25 min): One leg stand, stepping, dual task walking *Cooling down:* Parachute, stretching.	Themes: Ordinary training as volunteers: 1. Information about being a volunteer. 2. The role as volunteers in this project. 3. Dementia and consequences of the disease. 4. Communication and working together. 5. Meeting a volunteer (experiences from being a volunteer). Specific topics in this project: 6. Presentation of the exercise programme by the Physiotherapists.

*The rationale for the weekly group-based exercise is the ACSM Physical Activity Recommendations, the content is inspired of the “Strong and Steady” exercise concept in Norway, which is based on the OTAGO and FaME concepts, from Australia and the United Kingdom [20–23]

### Recruitment

Four older adults who were referred to the geriatric outpatient clinic, St Olav Hospital, University Hospital of Trondheim, for a cognitive evaluation were enrolled in this study. The responsible medical doctor who identified potential participants from the geriatric outpatient clinic informed the researcher, who then asked them about participation at the outpatient clinic at the end of their evaluation. The inclusion criteria for the present study was being home-dwelling, 65 years or older, with cognitive impairment or dementia, able to walk 10 m without walking aids or support, and wanting to take part in group exercise, once a week, to maintain their physical function. We excluded people with medical contraindications for exercise, evaluated by the medical doctor at the outpatient clinic (including serious or unstable systemic disease), and those who already took part in organised exercise twice a week or more. We also recruited their caregivers, if they had any, to take part in focus group or individual interviews. During the continuous recruitment, five potential participants did not meet our criteria and three eligible persons declined participation.

The counselling service centre, for people with dementia and their families, recruited five volunteers in total. Four of them attended a course for new volunteers in the municipality, including an extra session with the physiotherapist to get information about the group exercise programme and the trial. The responsible person at the counselling service centre matched the volunteers with the older adults that participated in the group exercises, based common interests, after performing home visits to the participants and interviewing the volunteers.

### Participants

From the exercise group consisting of four older adults and five volunteers, we included four older adults (M1, M2, F1, and F2) and their caregivers (C1–4), four volunteers (V1–4), and the two female physiotherapists (PT1 and PT2) that led the group exercises. All attended either a focus group or individual interviews. Table 2 describes the characteristics of the older adults recruited for the group exercises in this study, and their relation to the caregivers and volunteers. All the four caregivers who took part in interviews were women. Their relationships with the older adults were spouses (*n* = 2) or children (n = 2). The caregivers were not directly involved in the organisation/conduction of the group exercise training, but most of them did take part in inclusion and the matching session in their homes. The exercise period started with two older adults and three volunteers enrolled the first week, and we reached a full group at week four, with four older adults and five volunteers enrolled. We did not have any dropouts during the exercise period.

**Table 2:** Characteristics of the older adults taking part in the exercise groups (*n* = 4)

	Male (M1)	Male (M2)	Female (F1)	Female (F2)
Age, years	76	70	85	76
Gender	Male	Male	Female	Female
Living status/arrangement	With wife without paid help	With wife with help (practical home care and home nursing care)	Alone without paid help	Alone with help (home nursing care)
Cognitive function (CDR score)	1	1	0.5	1
Cognitive function (MMSE score 0–30)	25	24	24	19
I-ADL function (score 0–66)	45	48	60	58
Physical function (SPPB score 0–12)	11	11	12	11
Gait speed (m/sec) before start	0.91	0.73	1.18	0.89
Gait speed (m/sec) at final session	1.21	1.03	1.14	1.18
Sessions attended	12/12	11/12	7/10	6/8
Relationship to caregiver	**Spouse (C1)**	**Spouse (C2)**	**Daughter (C3)**	**Daughter (C4)**^a^
Matched volunteer	**Female student (V1)**	**Male (V2), Female (V3)**^b^	**Female (V4)**	^c^

*CDR* Clinical Dementia Rating score [24], *MMSE* Mini Mental Status Examination (MMSE) [25], *I-ADL-function* Nottingham E-ADL Index [26], *Physical function* The Short Physical Performance Battery (SPPB) [27] ^a^did respond via email due to not being available for an interview; ^b^V2 and V3 were a couple, both supported M2; ^c^the volunteer matched with F2 was not available to attend the interview.

### Interview setting

We conducted the focus groups and individual interviews either at the university hospital or in the municipality, with one of the individual interviews being performed in the participant’s home. All were performed in closed rooms without any interruptions. To ease transportation issues, we invited the older adults and their caregivers to their separate interviews on the same day. We completed two individual interviews and one group interview with the older adults, and one individual and one group interview with caregivers. We conducted a focus group interview with the volunteers, and we interviewed the two physiotherapists together.

### Focus group and interview procedure

The intervention period ran from March 2017 to early June 2017. All interviews were performed in June 2017, after cessation of group exercise programme.

An interview guide (see Additional file 1, Supplementary information) was developed based on literature and our previous study [16] to guide the discussions in all interviews. All participants were given time and opportunity to ask questions, both prior to starting the interview and after ending the interview. All interviews were audio-recorded, and one researcher transcribed verbatim. The audio-recorded data were checked by a second researcher, prior to performing any analysis.

The interview questions were designed to be open ended. Topics included why they took part and their experiences with the group exercise programme; the role of the volunteer/participant; the organisation; and their views on important factors to include when organising such services. We had similar topics for all participants, but the questions were phrased differently for the older adults participating, their caregivers, volunteers, and physiotherapist. If needed, the participants were given prompts and help, so that their experiences could be explored. Experienced researchers from the university who were not involved in the delivery of the group exercises performed the interviews, with duration between 30 and 60 min.

### Data analysis

NVivo 8 was used for systematising the data material, and the data were then analysed by Systematic Text Condensation method (STC) [28]. To get a general sense of the data set, and in line with the STC method, two of the authors read the transcripts independently. After discussing the overall impression, the next step was to identify meaning units. In this study, we defined meaning units as text fragments reflecting information about the participants’ experience with attending the volunteer-supported group exercise programme. The authors started coding by identifying and sorting meaning units. During the phase of de-contextualising, the authors reflected on the similarities and differences of each code. Based on a consensus after reading the transcripts of all interviews, the two authors agreed on the final codes. The first and the last author coded the interviews into themes and discussed them before they were presented to a group of the participants. Present at this meeting were, in total, eight participants: older adults, caregivers, and volunteers, but none of the physiotherapists. The two researchers that conducted the interviews led the meeting, and the responsible person from the counselling centre, who acted as a coordinator during the pilot, attended as observer only. We had a second meeting for representatives from the municipality responsible for similar services, including the two physiotherapists that participated in the study. We discussed the results with those who attended these meetings, to check our findings and gain input before the final themes were defined. These meetings confirmed the themes presented, and no changes were made to the final topics/themes. The last author then selected the quotes most illustrative for the chosen themes.

### Ethics

The Central Norway Regional Committee for Medical and Health Research Ethics approved the study (REK 2016/1603). All persons with dementia signed an informed consent form prior to starting the exercise period. The other participants in the interviews signed informed consent forms before starting the interviews. The group exercises pilot was conducted within the Municipalities services, following safety instructions and guidelines in line with other group exercise services. Information to all participants was given orally at all stages in this study.

## Results

### Topics/themes

All participants and caregivers expressed clearly that they had a positive experience with this group exercise pilot, both as participants in the exercise groups and as caregivers to the participants. All expressed a wish to continue, with participants wanting to take part in such group exercise programme and caregivers wanting their spouse/parent to continue, should this become a permanent offer after the study period.

The topics and meaning units from the data analysis are presented in Table 3.

**Table 3:** Topics/themes and meaning units

Themes	Meaning Units
**Building relationships**	**Code 1) Sense of belonging** **Code 2) Equality** **Code 3) A very good atmosphere**
**Support and organisation**	**Code 4) A feeling of safety** **Code 5) Common interests** **Code 6) Gaining experience and knowledge** **Code 7) Providing support and social interaction**
**Motivation for participation in activities outside home**	**Code 8) Importance of social time** **Code 9) Relief for relatives**

### Building relationships

The group exercise we tested in this pilot study was reported as important, due to creating a social arena for the older adults where they felt they belonged. Participants got to know each other and enjoyed being part of this group. As reported by two of the volunteers: *“Yes, we had coffee after each session. It has been very good. We are talking about different things, and we get to know each other very well.” (V2),* and *“I became very positive to the concept. As you said, I think it meant a lot for them. They have looked forward to this. “See you next week” and…”(V1).*

As stated by one of the older adults: *“… everything was ok, and then we had…we had coffee afterwards.” (M2)*.

The group was relatively small and stable, the volunteers were matched with the older adults, and together this led to a feeling of belonging, for both parties. As illustrated by these comments from two of the older adults: *“…It was like that you looked forward to start” (M1)*, *“Yes, indeed, they are so nice all of them.” (F2),* and this comment from one of the volunteers:“ *It is more fun to sit and talk to someone, rather than go for a walk and not meeting anyone.” (V3)*. One of the volunteers confirmed the feeling of belonging as illustrated with this comment:“ *I did think that we would get to know the persons, talk to them, or to the person, and become like a small community, that was what I thought, but I did not know that it was going to be as it was, because we did get very close, so I was positively surprised!” (V1)*.

Caregivers confirmed that they thought that being part of the group was positive for the older adults because they belonged in this group, as one daughter described:" *I think my mother found the whole experience fun. They were together and did exercise together. She told me: “we do a lot of nonsense” she said, “we laugh a lot”, she said." (C3)*.

Secondly, the caregivers and the physiotherapist highlighted the equality in the group as important for building relationships. As described by one of the physiotherapists:" *I felt that they were equal participants in this group. Not an “us” and “them”, but a “we” as one group. I think this strengthened the community in the group. The volunteers supported all participants in the group, not limited to the one person they had special" (PT1)*. This was also confirmed by the caregivers, as one described:" *In that way, it has been surprisingly positive for me as well, because he has had someone to talk to. He has told me about the student and, yes not about the exercise, but...talked about your husband also, a lot, so it is very clear that…" (C1)*.

Thirdly, the atmosphere in the group was highlighted as important by older adults, volunteers and caregivers, as illustrated by these two quotes: *“We had a very good atmosphere in the group.” (M1)*, and *“I believe that it has been that in the whole group. I think this is important, that you have a good mood and can joke a bit” (V3)*. The group atmosphere was safe, and they felt that they could joke and have fun in this group.

### Support and organisation

The need for sufficient support and organisation for this group exercise was highlighted by the volunteers and the physiotherapists, because they felt this was an important element of the positive experiences reported. Firstly, they reported that the feeling of confidence was important for the older adults, because they knew each other in this small and settled group, as illustrated by these quotes: *“Yes, the numbers and how settled it is … … we had a fixed sample who attended every session. It became very safe because you get familiar with them. A big group with random attenders, where there is a big exchange of people, is followed by more insecurity” (PT1)*, *“I thought that it was going to be more like just being with that person, being just that, but it has not been like that, because it has been a group in some way.” (V1)*, and“ *...the participants did have a high function, but I think they felt safety knowing they got to meet the volunteers prior to starting in the group, they knew who each other were, and «ok, you will come and pick me up» then everything was ok for them.” (PT1)*.

The volunteers also reported that common interests were important for getting to know each other and enjoying being together, as illustrated with this quote: *“Yes, it is about the importance of common interests. I have been a volunteer before where there was not a match between us. Then it felt like a duty.” (V4)*.

Furthermore, the volunteers highlighted that they gained experience and knowledge by being part of this project. The volunteers got to know the older adults with cognitive decline and dementia and found it educational, as illustrated by these two quotes: *“My wife and I did agree that we would both join, one as the driver and one as the active part matched with a person with dementia…If one was not available, the other could do it. It did work very well…after a while I found it educational. I also found it like a win-win-situation, where you do get exercise yourself…and after a while we did get to know our (participant), and then we found it very nice, especially him…the person we have picked up every time, he’s got a lot of knowledge, and it has become very nice, indeed. (V3)* and" *First, I am a social person, so it gave me something without any costs, something that others might appreciate. At the same time, we had a lot to talk about, as I said earlier, he is, − we cannot notice that he has dementia, he might be a bit forgetful, forgetting a word or something, but not more than what I or my wife forget. I looked forward to Thursdays. Actually.” (V3)*.

The volunteers and the physiotherapists reported that the support for social interaction was an important task for the volunteers within the group exercise session. As one physiotherapist described: “*I think the group dynamic differs by using volunteers. They talked with them. It was rarely conversations between the persons with dementia, so the volunteers kept the conversations going” (PT1)*, and confirmed by one of the volunteers: *“I think the social part is one of the most important tasks” (V2)*.

### Motivation for participation in activities outside home

All of the older adults highlighted the importance of social time that ensured motivation to take part in activities outside their homes, as two of themsaid: *“It was fun.” (M1)*, and *“Yes, it was fun”. (M2).* The caregivers confirmed this, that they thought it was important for the older adults to have something outside home to be part of, as illustrated with these comments: “*Yes, because my husband is rather lonely, he does not meet many persons, so this is what he now has attended, and I do think it means something for him (C2)*,” *It is something about that they have something, something that happens, I think it is both the experience of having the joy of exercising, but also that it is social interaction with someone else." (C2)*, and" *I think it is very positive, because my husband has said that he thinks it is very nice to sit and have a coffee together and talk" (C2)*. In addition to the exercise, the coffee afterwards was equally important, as commented on by one of the volunteers: *“Yes, I think it has been very positive, and I think they (older adults) also agree, both in terms of exercise, but quite as well the coffee they get afterwards, that was the highlight of the day, I think. (V2).* This was also underlined by one of the physiotherapists, who said that because many are getting more isolated, motivation is a key factor to get them to take part:” *What gives motivation is not important, as long as you manage to get started" (PT2).*

For the caregivers, this group exercise was a relief in terms of having something outside home to be part of, as described by one of the volunteers: “*I think especially for one of the men who lives with his wife, that she thinks that it is very ok that he is going out on his own” (V2)*.

## Discussion

We tested a 12-week, group-based exercise intervention delivered by two physiotherapists with support from volunteers. Through interviews with all four older adults taking part in the exercise groups, their caregivers, four of the five volunteers, and the two physiotherapists, we wanted to understand their perceptions of the feasibility and impact of this group exercise. Overall, we found that this new offer was a success in terms of engagement and appeal. There was no drop out in the pilot study, with all older adults and volunteers continuing participation until the study had ended, and expressing that they wanted this to be a permanent service. Three themes, one main and two supplementary, emerged in the focus groups and interviews,

The main theme that emerged was the importance of building relationships between those attending the exercise group. The exercise group created a new social arena for the persons with dementia, and they felt a strong attachment to the group. We found that equality between the older adults and volunteers in the group was highlighted by our caregivers and physiotherapists in terms of being able to build relationships. The feeling of equality could be a result of getting to know each other. Getting to know each other as individuals was more important than knowledge about the older adults’ diagnosis of dementia, and possibly also more important than the content of the exercise programme. Furthermore, they felt the atmosphere in the group as positive, and it felt safe to joke and have fun because of this. These results suggest that building relationships should be of high importance when offering new services for this target group. Exercise offers are often organised and planned without this in mind, although the feeling of belonging emerged as the key to the feasibility of the service offered in this study. Another study confirming this describes this as “fellowship” where the participants are enjoying the activities because they are included and are able to be themselves [29].

Behind the overall positive experience of this new offer tested in our study, one theme that emerged when talking to the volunteers and the physiotherapists was the importance of support and organisation. Support to make sure older adults feel safe in the group is highly important. To be able to achieve this, all participants in such services need to know each other. Having relatively small and stable groups seems important. Furthermore, the volunteers reported common interests with the older adults as important; they knew each other and enjoyed being together. Our results are in line with research on behaviour change theories, suggesting that individuals with dementia need extra support both to be able to start and continue with new activities [3]. Thus, identifying factors that influence adherence to exercise is crucial and should be included in development of interventions for this population [30]. The volunteers said they learned a lot from being part of this project, where the dementia diagnosis was not in focus, but rather the interesting person behind the diagnosis. Organisation of this service, by use of volunteers, was positively evaluated, with the volunteers having an important role for the social interaction in the group. These results could be interpreted as “care support” that Sutcliffe et al. (2015) describe, where support services appropriate to the caregivers’, and their relatives with dementia’s, needs are important for success [31]. This study also confirms that regular staff members in services is important, because staff changes or absenteeism create challenges in many services. Our results are in line with Sutcliffe et al. (2015), where single point of contact and continuity should be integrated in services for persons with dementia [31].

The final theme that emerged in our study was motivation for participation in activities outside the home. Results are in line with previous work underlining the importance of participating in activities [32, 33]. Motivation to take part in group activities is essential for both starting and continuing to attend. Our results indicate that the social interaction was the main motivation to continue to take part in the exercise class. Caregivers felt it was essential that the participants had this offer outside the home to participate in. In addition, this service supported them as caregivers, which is in line with previously reported suggestions from caregivers [16]. The potential effects of maintaining physical activity and physical function was the reason for choosing exercise as activity in this service. We do not know from our results if the older adults, volunteers, caregivers, or the physiotherapists who took part in our study thought that the activity in such services must be exercise, but in line with other exercise pilots [7], exercise interventions could be feasible and show enjoyment. This is confirmed by the results indicating that the participants in our pilot had fun while exercising, and that they experienced this service as positive. From other work, fun is shown to be an important motivator for older adults for engaging in PA and exercise [34].

Caregivers of people with cognitive decline and dementia often experience a high burden or stress, often related to their experiences with many services as a “struggle” or “uphill fight” [31]. Services that motivate participants to participate outside their homes are therefore highly important. One study evaluating family caregivers’ management of behavioural and psychological symptoms of dementia suggest strategies such as activity engagement, maintaining a sense of humour, social support, and taking time off from being caregivers, among others [33]. In line with this our trial showed that participation was important due to providing a supportive social arena for our participants. They felt safe, could joke and have fun, all which are important for their overall health, as well as providing an important break for the caregivers.

This study has some important limitations. First, we recruited home-dwelling participants from the geriatric outpatient clinic who had mild dementia or mild cognitive impairment, meaning that we do not know if this model can be applied among persons in more advanced disease stages. Furthermore, we only included participants with caregivers. Inclusion of persons with cognitive decline or dementia without caregivers could potentially be more challenging. We did manage to include both men and women, and their caregivers were wives and daughters. Although we recruited from those already in contact with the health care system, we believe our target group is an important group to reach, especially because one criterion was not attending other group activities outside their homes.

This was a small pilot study, and more knowledge is needed on how to develop and run such group exercise interventions for persons with dementia. The overall positive feedback from the experiences from all participants involved in this pilot can however provide useful guidance, especially for further development of such interventions in the future. Our small group of participants were those who wanted to join the exercise pilot, and our findings may not provide insight into all factors important for running such exercise services, especially in terms of recruitment strategies on larger scales. The different views from older adults, as well as their caregivers, the volunteers and physiotherapists, strengthens the results and provides important knowledge into factors important for the development of new services for this target group of older adults with dementia. More knowledge on how to recruit people with dementia, also those without caregivers present, should be explored further in future research.

## Conclusion

Our 12-week, group-based exercise intervention for community-dwelling older adults with mild cognitive impairment and dementia was experienced as very important for the participants. We have gained perceptions from older adults, caregivers, volunteers, and the two physiotherapists leading the group sessions, with all reporting that building relationships was the most important factor for the success of this group exercise. Along with the importance of getting people to feel that they belonged in the group, support and organisation, as well as motivation to take part in activities outside home, are factors we should address to be able to get people involved in such services. This study has come up with factors to be considered and included in the planning of group offers, in order to be successful in organising group offers for persons with cognitive decline and dementia, and for similar groups where participation in activities outside their homes may be a challenge.

## Supplementary information

**Additional file 1.**

## Data Availability

The datasets generated and analysed during the current study are not publicly available at this stage.

## Abbreviations

ADL: Activities of daily living
PA: Physical activity
