# Supplementary material for: Client, caregiver, volunteer, and therapist views on a voluntary supported group exercise programme for older adults with dementia
Source: BMC Geriatr. 2020 Jul 8;20:235. doi: 10.1186/s12877-020-01632-6 (PMC7346355; doi:10.1186/s12877-020-01632-6)
Supplement: Supplementary file 1 — Additional file 1. [file 12877_2020_1632_MOESM1_ESM.docx]

**Qualitative questions after completing the exercise period (note: we only translated to overall questions and not the prompts/examples)**

**QUESTIONS TO OLDER ADULTS:**

**1. First, can you tell us a bit about why you wanted to be part of this exercise intervention offer?**

**2. Can you tell us how you found being participant in the group exercise programme?***Prompt: What did you like? What did work? What was less fun? What should be changed?*

**3. You have a volunteer, who also attended the group exercises. How did you find the collaboration with the volunteers?**

*Prompt: What has been important in terms of being a “good match”? What kind of role did your volunteer have? (Prior to the group exercise, during the group exercise, after the exercise session.) How important was the volunteer for you and your participation in the group? Do you have any suggestions for changes?*

**4. You attended the whole project and participated in a lot of the exercise sessions, we wonder if you could tell us what you especially liked and why?**

*Prompt: Anything you missed? Anything we should have included more in the exercise sessions?*

**5. How did you find the physiotherapists leading the exercise sessions?***Prompt: What about the instructions (clear?)? Did you think that they acknowledged you? Did you get the support you needed? Did you get answers to your questions? Do you have any suggestions for improvements?*

**6. If this was to become a permanent offer, would you continue to take part?**

**7. Do you have any plans to join other exercise offers now, after this period with the group exercises?**

**8. What is crucial for you; what is important if you should accept to join a similar exercise offer once a week?**

**9. Do you have any other input that you think is important for us, related to how to improve such offers?**

**QUESTIONS TO CAREGIVERS:**

**1. First, can you tell us who you are caregivers for? (The person attending the exercise offer.)**

**2. What is your view on what was important for them when accepting to be part in this exercise offer?**

**3. Can you tell us a bit about what they have told you about the exercise sessions?**

*Prompt: Do you know what did work? What did not work? Why do you think they took part?*

**2. What do you think about the organisation of this offer?**

*Prompt: What did work? What did you miss? Do you have any tips when planning similar offers?*

**3. How did you find the matching with your husband/mother and the volunteers, and how did you find the contact between them/how did that work?**

*Prompt: Do you know if there was a good relationship? What did work? Do you find it necessary to have a person that can follow them to and back from the sessions? Do you think we should have planned/organised anything differently? Did your mother/husband talk about their volunteer – if yes, what did they tell you?*

**4. Do you have any suggestions for how we can improve such offers?**

**5. Your mother/husband was asked to attend this study when you attended the geriatric outpatient clinic at the Hospital. How was it for you and your mother/husband to be asked this question on this particular day? Looking back, what do you now think about recruitment for such offers?**

*Prompt: Where should recruitment take place/who should ask the question/when (according to functional level or time of diagnosis)?*

**6. Any other input?**

**QUESTIONS TO VOLUNTEERS:**

**1. First, can you tell us a bit about why you wanted to participate as volunteers in this exercise intervention offer?**

**2. Can you tell us what you think about the group exercise offer that you have been part of?**

*Prompt: What did you like? What did work? What did you not like? What should be done differently?*

**2. Was the offer as you imagined?**

*Prompt: What differed?*

**2. How did you find the organisation of this exercise offer?**

*Prompt: Anything we should have organised differently? What was your responsibility during the exercise offer?*

**3. How did the matching go between you and the person you supported? How did you find the training you received?**

*Prompt: Did you miss anything? What did work? Anything you should have learned/known more about, or was anything redundant? Could you say what in the training that did motivate you to attend?*

**4. If this was to become a permanent offer later, what do you think are the most important factors for the volunteers that will be involved in such offers, in terms of information/knowledge?**

**5. How did you find the exercise sessions? In what extent were the exercise sessions relevant for you?**

*Prompt: What was your primary task during the exercise sessions?*

**6. Do you have any other input to how to improve such offers?**

**7. How should we recruit volunteers in such offers – from where and through whom?**

**8. Would you consider to continue being part in such an offer?**

**9. Do you have any other input that you think is important for us, related to how to improve such offers?**

**QUESTIONS TO PHYSIOTHERAPISTS:**

**1. First, what kind of experience did you have with the target group prior to taking part in this pilot?**

*Prompt: Is this something quite new for you, or did you have any knowledge/experience from before? Can you give examples of similar offers that you have been part of, or other?*

**2. What do you think was the main focus in the exercise sessions?**

*Prompt: What was the starting point? At the end, did it turn out as you thought/had planned?*

**3. What adaptions were necessary?**

*Prompt: Did it turn out to be different than expected? In any case, what was as expected and what did differ? Did you get any surprises during this period?*

**4. What do you think is important when planning and conducting such offers, and why?**

*Prompt: Is there something that you as physiotherapists need to have knowledge about, prior to starting such offers to a group with dementia?*

**5. What would an optimal offer look like, as compared to what you have been part of now?**

*Prompt: What would be similar to this offer / what would be different?*

**5. After ending the exercise period – what do you now think is important and do you have any input that is relevant for improving such offers?**

**6. What is important for success? (For participants to want to joint, that they experience it as fun and purposeful.)**

**7. Any other input?**
